# Optimizing Operation Time and Travel Distance for Motorcycle Ambulances in Emergency Medical Services

**DOI:** 10.1017/S1049023X2200228X

**Published:** 2023-02

**Authors:** Korakot Apiratwarakul, Pariwat Phungoen, Lap Woon Cheung, Somsak Tiamkao, Takaaki Suzuki, Chatkhane Pearkao, Kamonwon Ienghong

**Affiliations:** 1. Department of Emergency Medicine, Faculty of Medicine, Khon Kaen University, Khon Kaen, Thailand; 2. Accident & Emergency Department, Princess Margaret Hospital, Kowloon, Hong Kong; 3. Emergency Medicine Unit, Li Ka Shing Faculty of Medicine, The University of Hong Kong, Pokfulam, Hong Kong; 4. Department of Medicine, Faculty of Medicine, Khon Kaen University, Khon Kaen, Thailand; 5. Department of Emergency and Critical Care Medicine, University of Tsukuba Hospital, Tsukuba, Japan; 6. Department of Adult Nursing, Faculty of Nursing, Khon Kaen University, Khon Kaen, Thailand

**Keywords:** Emergency Medical Services, motorcycles, prehospital emergency care, response time, transportation

## Abstract

**Introduction:**

The motorcycle ambulance is used for quick access to patients. The response time to reach the patient takes less time than with a van ambulance. Moreover, accidents involving ambulances tend to be higher. However, at present, there is no study regarding the appropriate situation used of motorcycle ambulances in Emergency Medical Services (EMS) in Thailand.

**Study Objective:**

This study aims to optimize the travel distance and the operation time of motorcycle ambulances used.

**Methods:**

This study was a prospective, randomized controlled study at the EMS unit of Srinagarind Hospital, Thailand. The data collection period was from November 2021 through May 2022. All data involving dispatch of both ambulances in need were collected.

**Results:**

A total of 2,398 cases of EMS operation were examined. The mean age of the patients in the motorcycle ambulance group was 42.5 (SD = 6.5) years, and 51.3% (n = 616) were male. The response time for motorcycle ambulances and van ambulances during the operation time between 6:00am-9:00am was 6.2 minutes and 9.1 minutes, respectively. The response times for motorcycle ambulances and van ambulances regarding distance traveled from 0-5km were 4.2 minutes and 7.5 minutes, respectively (P <.001); distance traveled from 5-10km were 6.3 minutes and 8.2 minutes, respectively (P = .010).

**Conclusion:**

The motorcycle ambulance can reach patients faster than the ambulance at the operation time from 6:00am-9:00am and 3:00pm-6:00pm. This study focused on the distance less than 10 kilometers.

## Introduction

Thailand’s Emergency Medical Services (EMS) were established by and are overseen by the National Institute for Emergency Medicine (NIEM; Nonthaburi, Thailand), which determines operating standards in terms of medical equipment, vehicles, and qualifications of operators in EMS.^1^ There are six stages in the EMS process: detection, reporting, response, on-scene care, care in transit, and transfer to definitive care. Every step is important, especially at the on-scene care stage which the length of time to reach the patient plays an important role for providing the initial assessment and the treatment of prehospital patients. Personnel working in the EMS include emergency physicians (EPs), emergency nurse practitioners (ENPs), registered nurses (RNs), advanced emergency medical technicians (AEMTs), and emergency medical technicians (EMTs). The type of ambulance used to access the prehospital patients regulated by NIEM can be categorized as ambulance Type I and Type II, which is characteristic of pickup trucks and vans. However, the limitation of these vehicles was the traffic and road conditions, especially during rush hours in large cities, which made delays in gaining access to emergency patients. To solve these problems, a new type of ambulance used a motorcycle model designed for access through congested areas, constrained spaces, or difficult terrain much more quickly and easily than bigger four-wheeled vehicles.^2,3^

The model of motorcycle ambulance has been developed sequentially, initially being used in African countries with limited resources to transfer the patient from the community hospital into a central hospital by focusing on delivering patients with problems in obstetrics and gynecology. The use of this vehicle aimed to transfer pregnant women with labor period from rural areas to a hospital which manages this condition. The driver of the motorcycle ambulance was a volunteer in the public health system who does not train for emergency conditions. The design of motorcycle ambulance was still a factory model, with no medical equipment attachment. From previous studies,^4,5^ it was found that the response time to reach the patient and the transport time to deliver the patient took a shorter amount of time than an ambulance in the form of a van, and this led to a decrease in the expense of the operation.

In later periods, motorcycle ambulances were used to solve the problem of delays due to traffic congestion, especially in large urban areas. The study about the ambulance motorcycle in Africa^6^ found that the time it takes to reach a patient with a motorcycle ambulance is considerably shorter than that of a van or pickup ambulance. Moreover, this type of ambulance also has a wider range of places which it can travel. The condition of small road alleys has increased flexibility in accessing operations due to its smaller size, such as alleys and narrow pathways. Nowadays, the model of motorcycle ambulance has progressed far from the initial development. The automated external defibrillator (AED) was added to the new model of motorcycle ambulances, since in developing countries, it is difficult to install AEDs in public spaces due to a lack of funding for the procurement of the equipment. Nevertheless, the people’s understanding on how to utilize the technology is also lacking. When compared to van ambulances, this new model has shorter response times, which increases the number of continuous resuscitations in out-of-hospital cardiac arrest patients.^7^ However, a motorcycle ambulance also had some disadvantages, such as it cannot operate in heavy rain conditions or a severe windstorm. This may result in danger to the EMS members.

The command-and-control centers that dispatch EMS units operating in Thailand rely on taking calls through the emergency phone number “1669.” The dispatcher assesses the patient’s condition and determines the level of EMS operation into three levels, including red, yellow, and green, according to the severity of symptoms.^8,9^ The red level being a critically ill patient who urgently needs access, in which case the motorcycle ambulance would be the optimal choice. The factors which affected the prehospital time were the number of EMS operating orders, parking locations for ambulance vehicles, the season, the traffic and road conditions of major cities, and the demand of non-conveyance patients used in EMS operating systems.^10–13^

Previous studies^6,7^ have demonstrated that a motorcycle ambulance is beneficial in terms of the quick access to the patients, moving into limited spaces, and prompt providing of medical equipment. There are, however, limitations to the operation of motorcycle ambulances, such as operating in rainy weather or a severe windstorm. This carries a risk for motorcycle ambulance operators. It has been shown in many studies carried out in many countries that accidents involving ambulances tend to be higher, and there was much more severe injury to body, life, and property.^14–16^

Therefore, it is crucial to select the distance and best operation hours when using motorcycle ambulances. However, no research on the advantages of employing motorcycle ambulances has been conducted. In order to decrease the response time vehicles take to arrive and increase overall driver safety, this study aimed to identify optimal distance from hospital to scene and the suitable operation time used for motorcycle ambulances.

## Methods

### Design and Setting

This study was a prospective, randomized controlled study at the EMS unit of Srinagarind Hospital, Faculty of Medicine, Khon Kaen University, Thailand. The emergency unit has five van ambulances, together with one motorcycle ambulance. There are on average more than 2,000 EMS operations per year. The level of operations was at the advanced level in approximately 1,000 cases per year.

The location of Srinagarind Hospital is located at Khon Kaen University in Khon Kaen Province, which lies 450 kilometers (km) northeastern of Bangkok, the capital of Thailand. Khon Kaen is one of the major cities in the northeastern region of Thailand. The administration of the city is approximately 46 sqkm.^17^ Khon Kaen population data in 2021 was 509,000.^18^

The study area was categorized as in and out of the university campus. In the distance below five kilometers from ambulance parking, the characteristic of the area was university campus. The campus road contained two-way traffic, the width of the road was small in size and varied from less than six meters to 12m in width (two-way two-lane, with set white line to separate the traffic flow on the road). The area of the campus is made up of the teaching office district, the life leisure district, the entertainment district, the centralism green zone, the logistic services area, and so on. The distance of more than five kilometers from the hospital, the street structure was the highway road named Mittraphap Road, the road linking Bangkok to the Thai Lao Friendship Bridge, which was two-way four-to-six lanes, with the island in the middle of the road. Moreover, there are many agricultural areas.

### Data Collection and Outcome Measurement

Data collection took place from November 2021 through May 2022. Participants were the patients who were attended to by both van ambulance and motorcycle ambulance in the study period. Cases with incomplete data, those that went out of EMS operation and did not find a patient, and those subjected to inclement weather were excluded from this study.

The process of EMS operations in Khon Kaen Province starts with emergency patient calls for help at the emergency services 1669 call center; the call taker and dispatcher will take a preliminary history to assess the severity of the patients and divide the patients into three categories (red, yellow, and green) following the Thailand emergency medical triage protocol and criteria-based dispatch.^19,20^ The dispatcher then orders the EMS operation. After that, the EMS unit of Srinagarind Hospital receives that order, and the EMS operation will begin.

In terms of the type of vehicles, the EMS operation in this study used motorcycle and van ambulance. Motorcycle ambulance^2^ was modified from the Honda New Forza 300 (Honda Motor Company, Ltd.; Tokyo, Japan) models to create the motorcycle ambulance.

The width of the model is 75.30-centimeters, length 216.60-centimeters, height 118.90-centimeters, seat height 71.60-centimeters, ground clearance 13.90-centimeters, and has a net weight of 192 kilograms. The engine had four strikes, one cylinder, and four valves. The fuel was gasoline 91, 95, or E20, with a fuel tank capacity of 11.5 liters.

**Figure 1.**
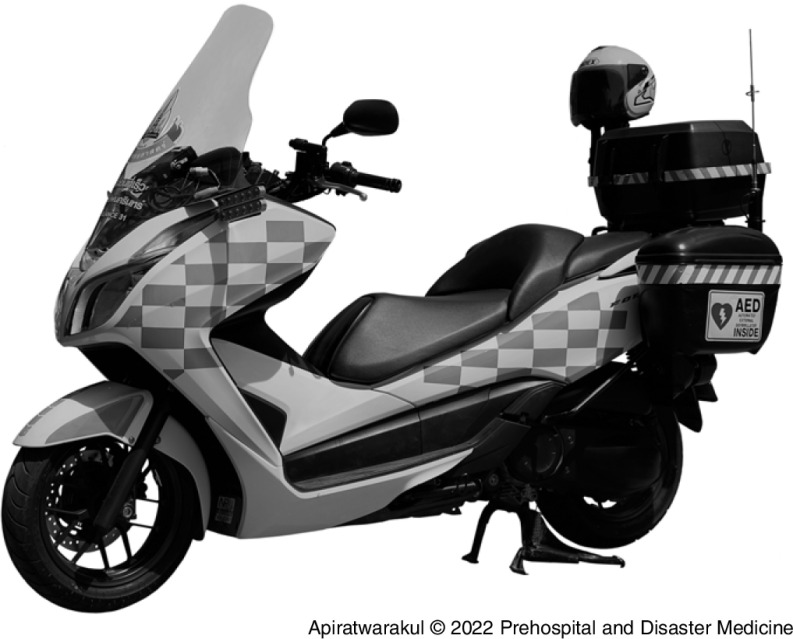
Srinagarind Motorcycle Ambulance.

The three device boxes were installed in the rear of the motorcycle ambulance, one on each side and one behind the passenger seat. The AED and airway management equipment were stored in a box attached to the side of the motorcycle ambulance for easy access (Figure 1).

The van ambulance was modified from the Toyota commuter model 2021 (Toyota Motor Company, Ltd.; Tokyo, Japan) to operation order by NIEM Thailand standard for EMS operation.

**Figure 2.**
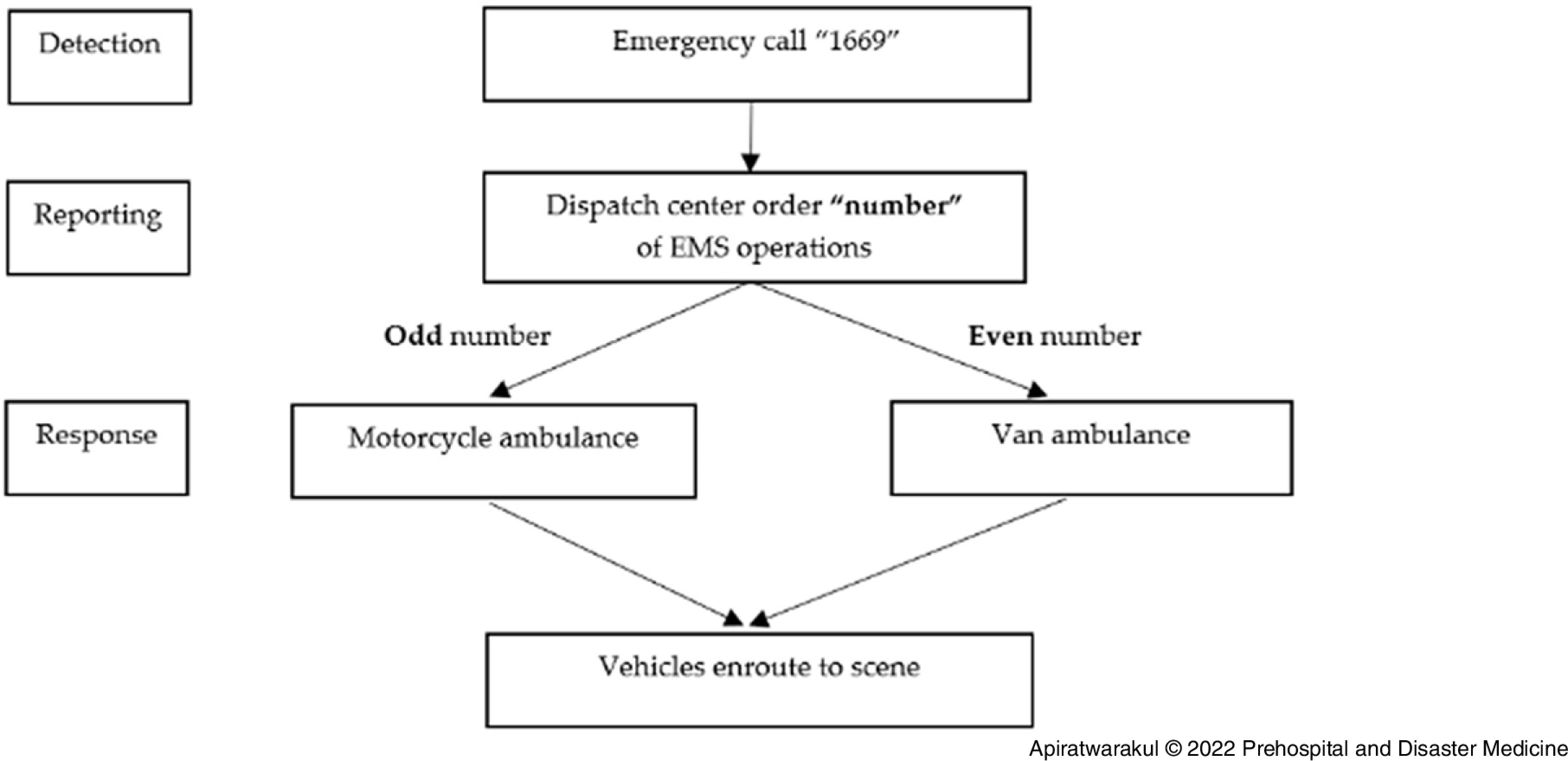
Algorithm of the Study. Abbreviation: EMS, Emergency Medical Services.

In terms of the randomization of vehicles, the study used standard EMS operation number provided from the 1669 center. The EMS unit of Srinagarind Hospital received the operation number, then categorized the number into two groups that were the odd number, which motorcycle ambulance was used for the operation, and in contrast, ambulance vans were used to operate in the even number (Figure 2).

Data were recorded using a national standard operation record form for Thailand EMS consisting of demographic data (age, gender), operation time, response time, type of patients (trauma, non-trauma), type of first procedure on scene, and physical distance of operations for all patients which were recorded at the EMS database of Srinagarind Hospital. The three independent, well-trained EPs extracted and reviewed these data from the EMS database. After that, all data were recorded twice in the Microsoft Office 365, KKU license (Microsoft Corp.; Redmond, Washington USA). The two data sets are then compared, differences are examined, and corrections are made.

The outcome of this study focused on the travel distance and the response time. First, the travel distance measurement from the hospital to the scene relies on the geographic coordinate system (GPS) with real-time telemedicine information to the command center installed on the van ambulance and motorcycle ambulance which parked at the same area, 20 meters from the emergency room area. The telemedicine system consisted of a central monitoring located in the dispatch center and five modular designs in each ambulance. The distance was calculated by GPS and displayed in digital number in kilometers at the EMS unit, Srinagarind Hospital. Second, the measurement of response time starts at the time when the 1669 center call is received and ends when the ambulance arrived at the scene, which was determined by one synchronized clock at the Srinagarind Hospital’s command center, the main clock used to store all data, displayed in digital format, and used Indochina time.

### Study Size

The sample size was calculated based on the previous study.^7^ The authors determined that a sample size of 1,200 would be required. The Khon Kaen University’s license for IBM SPSS for Windows version 27.0 was used for statistical analysis (IBM Corp.; Armonk, New York USA). Continuous data were presented using averages and standard deviations, whereas categorical data were given as percentages. A two-sample t-test for numerical data and a Chi-squared test for comparing data between groups were used in the univariate analysis. P value was identified as less than 0.05 demonstrated the significance of statistic.

### Ethical Considerations

The Declaration of Helsinki principles and Good Clinical Practice recommendations were followed throughout this investigation. The study was authorized by the Khon Kaen University Human Research Ethics Committee (HE641538). Because this study was based on ordinary EMS operations and patient confidentiality was assured, informed consent was waived. The patient’s identity could not be determined using the study number.

### Definition of Transport Metrics Used in This Study

Transport time was defined as the prehospital interval between departure from the starting location and arrival at the destination hospital. Response time was defined as the time from the 1669 center call received to arrival on the scene.

Dispatch time was defined as the time interval beginning with the time the initial 1669 phone call rings at the command-and-control center to dispatch EMS units requesting EMS, and ending with the dispatch time of the EMS unit responding.

Operation time was defined as the period that ambulance provided EMS services in each case. In this study, the time was divided into three-hour segments, starting at 6:00am and ending at 6:00am the following day.

## Results

In the seven-month period of study, a total of 2,398 cases of EMS operations were examined. The characteristics of the services and subjects are shown in Table 1. The mean age of the patients in the motorcycle ambulance group was 42.5 (SD = 6.5) years, and 51.3% (n = 616) were male. In both groups, the red level of criteria-based dispatches was the least common type of cases in the study. The type of patient in motorcycle and van groups mostly were in non-trauma type, 70.4% versus 66.5%, respectively. In first procedure on scene, the airway and breathing management were the highest numbers in both groups.

**Table 1.** Baseline Characteristics of Patients

Characteristics	Motorcycle Ambulance (N = 1,200)	Van Ambulance (N = 1,198)	*P* Value
Sex, N (%)			.701
Female	584 (48.7)	552 (46.1)	
Male	616 (51.3)	646 (53.9)	
Age (year) Mean (SD)	42.5 (SD = 6.5)	44.2 (SD = 8.1)	.652
Type of Patients, N (%)			.552
Trauma	355 (29.6)	401 (33.5)	
Non-Trauma	845 (70.4)	797 (66.5)	
First Procedure On Scene, N (%)			.442
Airway and Breathing	610 (50.8)	674 (56.3)	
Circulation	507 (42.3)	499 (41.7)	
AED Attachment	83 (6.9)	25 (2.0)	
Criteria-Based Dispatch Level, N (%)			.621
Red	105 (8.8)	118 (9.8)	
Yellow	520 (43.3)	533 (44.5)	
Green	575 (47.9)	547 (45.7)	

Abbreviation: AED, automated external defibrillator.

**Figure 3.**
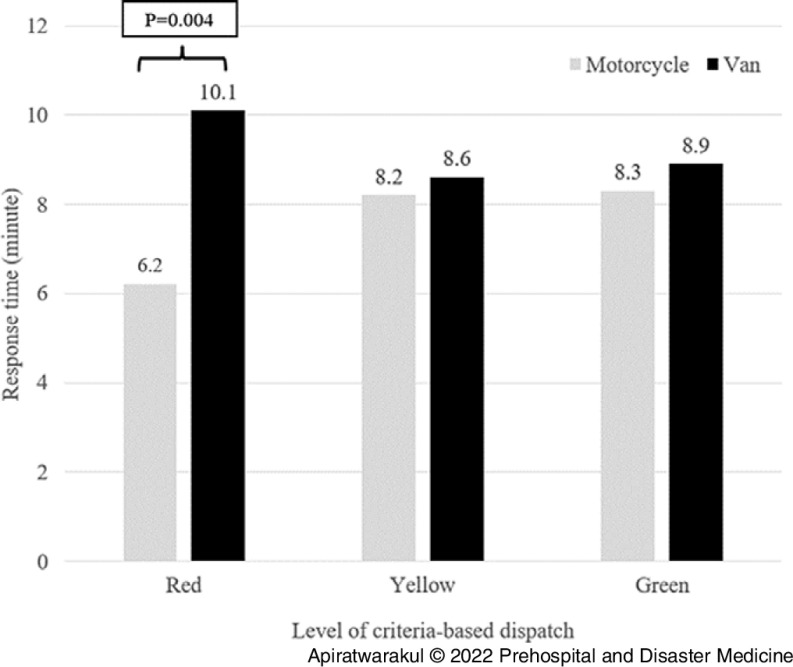
Response Time of Ambulances and Criteria-Based Dispatch Level in 06:00-09:00.

**Figure 4.**
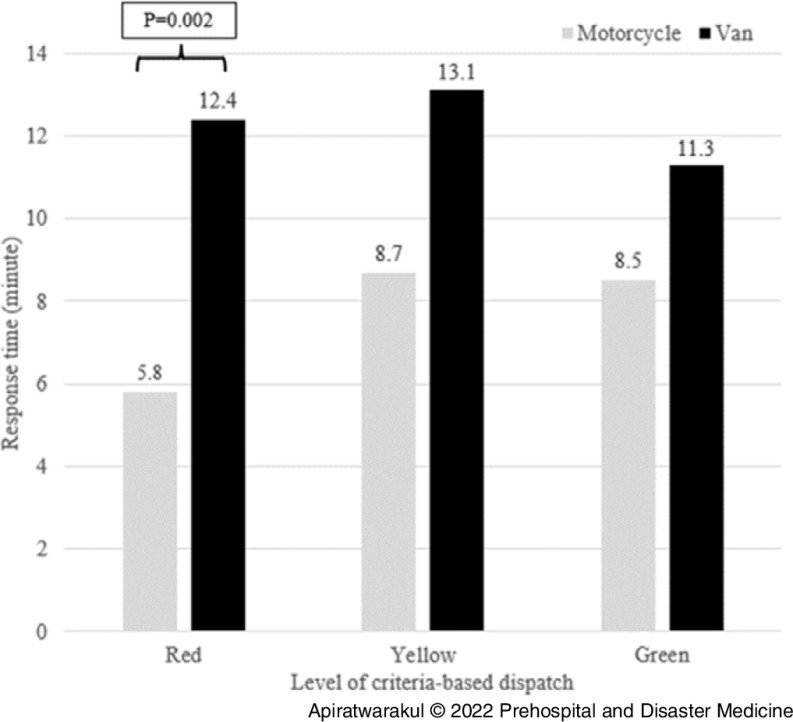
Response Time of Ambulances and Criteria-Based Dispatch Level in 15:00-18:00.

The response times for the motorcycle ambulance and the van ambulances in the period of 06:00-09:00 were 6.2 minutes and 9.1 minutes, respectively (P = .004; Table 2). During the 06:00-09:00 period, the red level of criteria-based dispatch for motorcycle ambulances used a shorter response time than van ambulances (P = .004; Figure 3). Additionally, for the period of 15:00-18:00, the red level of criteria-based dispatch for motorcycle ambulances used a shorter response time than van ambulances (P = .002; Figure 4).

**Table 2.** Response Time of Ambulance Operation in Each Period of Operation Time

Period of Operation Time	Motorcycle Ambulance (N = 1,200) (min) Mean (SD)	Van Ambulance (N = 1,198) (min) Mean (SD)	*P* Value
06:00-09:00	6.2 (SD = 2.1)	9.1 (SD = 1.1)	.004^a^
09:00-12:00	10.4 (SD = 4.6)	10.6 (SD = 5.0)	.201
12:00-15:00	10.5 (SD = 5.2)	10.8 (SD = 6.0)	.225
15:00-18:00	8.1 (SD = 2.3)	12.5 (SD = 2.1)	<.001^a^
18:00-21:00	11.2 (SD = 4.1)	11.6 (SD = 4.3)	.420
21:00-00:00	10.2 (SD = 2.3)	10.5 (SD = 2.5)	.422
00:00-03:00	6.2 (SD = 2.4)	6.4 (SD = 2.6)	.520
03:00-06:00	5.4 (SD = 4.2)	5.5 (SD = 4.3)	.510

a Statistically significant.

**Figure 5.**
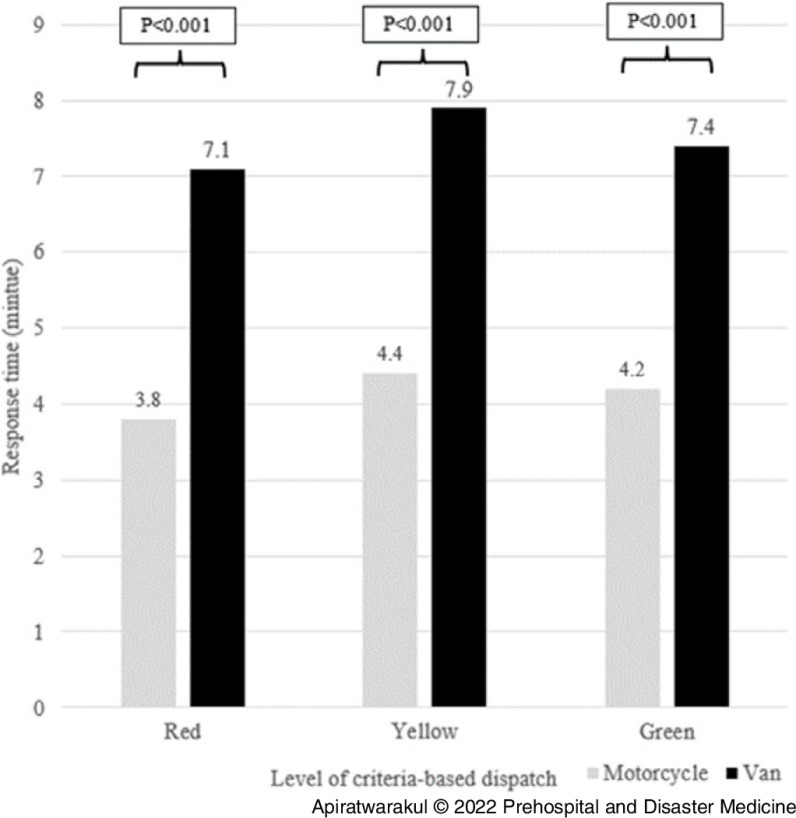
Response Time of Ambulances and Criteria-Based Dispatch Level in 0-5km.

**Figure 6.**
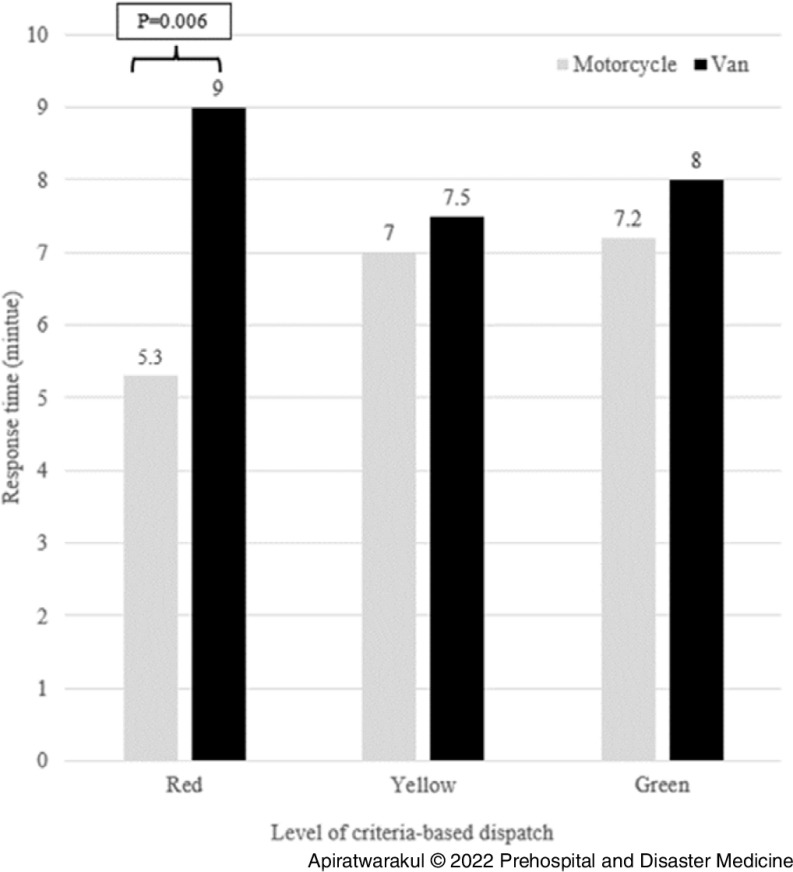
Response Time of Ambulances and Criteria-Based Dispatch Level in 5-10km.

In Table 3, the response times for the motorcycle ambulance and the van ambulances in distances from 0-5km were 4.2 minutes and 7.5 minutes, respectively (P <.001); for distances ranging between 5-10km, response times were 6.3 minutes and 8.2 minutes, respectively (P = .010). For the distance 0-5km, all level of criteria-based dispatch for motorcycle ambulance used a shorter response time than van ambulance (P < .001; Figure 5). At the 5-10km range, the red level of criteria-based dispatch for motorcycle ambulances used a shorter response time than van ambulances (P = .006; Figure 6).

**Table 3.** Response Time of Ambulance Operation in Each Distance of Operation

Distance	Motorcycle Ambulance (N = 1,200) (min) Mean (SD)	Van Ambulance (N = 1,198) (min) Mean (SD)	*P* Value
0-5km	4.2 (SD = 0.5)	7.5 (SD = 0.4)	<.001^a^
5-10km	6.3 (SD = 1.0)	8.2 (SD = 1.1)	.010^a^
10-15km	12.4 (SD = 1.6)	10.1 (SD = 2.0)	.520
>15km	20.2 (SD = 2.2)	16.2 (SD = 1.6)	.622

a Statistically significant.

## Discussion

This study aimed to analyze and compare the appropriate distance and EMS operation time for a motorcycle ambulance and compare it with that of a van ambulance operation. In a previous study, the motorcycle ambulance was shown to be useful in reaching emergency patients quickly, especially in large cities with traffic problems. However, ambulance crashes tend to be higher, and injuries sustained from the traffic accidents more severe. Therefore, a clear course of action must be established by selecting the appropriate operating time and distance, which are the objectives of this study.

In this study, the number of emergency patients operating in the EMS with criteria-based dispatch level by phone showed that urgent patients (yellow) and non-urgent patients (green) had the highest population, consistent with previous studies.^21,22^

In terms of operation time, motorcycle ambulances were found to reach patients quickly between 6:00am-9:00am and 3:00pm-6:00pm. Both periods are considered rush hours due to students traveling to and from school and employees traveling to and from work, resulting in the sharp rise in the number of vehicles on the road and frequent traffic jams.^23^ This causes serious delays in reaching the patient, so motorcycle ambulances play an important role in gaining more immediate access during that time. There are also cultural dimensions at play. In Thai society, most parents tend to travel to pick up and drop off the students at school regularly, in contrast with the Western countries in which students travel to and from school on their own.^24,25^

In addition, in a closer examination of the relationship between operation time and criteria-based dispatch level, it was found that motorcycle ambulances can reach patients with resuscitated level (red) from 6:00am-9:00am and 3:00pm-6:00pm faster than traditional van ambulances. This may be due to EMS personnel being trained to respond quickly in this type of patients; the example of patients categorized as red following criteria-based dispatch in this study were unresponsive patients, seizure, respiratory distress, shock, air-way obstruction, or major trauma, all which need immediate treatment. The standard operating criteria in unresponsive patients is to reach the AED as soon as possible. When the EMS crews are ordered to respond to unresponsive patients, EMS crews usually respond immediately to rapidly attach AED to those patients. Thus, the motorcycle equipped with AED has an advantage in this point. This was consistent with previous studies that focused on timing of access to defibrillators in patients with out-of-hospital cardiac arrest correlated with the survival rate.^26–28^

In terms of operating distance, it was found that motorcycle ambulances can reach patients faster than a traditional ambulance at distances below 10 kilometers. The reason for this is because a motorcycle ambulance can en route faster than a van. In addition, only one or two personnel on a motorcycle ambulance are required. This is different from the number of personnel operating on van ambulances, which require at least three personnel.^2,7^ Therefore, the time to start with a motorcycle is quicker. Another reason is the geography around the hospital in a 10-kilometer range. Most of these areas are roads with a small traffic surface, or an alley, making access by motorcycle possible, but not for a van, as it is highly maneuverable in confined spaces. In the distance range of more than 10km, it was found that motorcycle access takes longer than van ambulance access. This is due to the maximum speed of both vehicles and the road surface has four large traffic lanes. This allows a traditional ambulance to be able to use a higher speed than a motorcycle.^29^

In terms of the relationship between operating distance and criteria-based dispatch level, it was found that at less than five kilometers, motorcycles will reach patients faster than vans in all levels of severity (red, yellow, and green). This was due to the distance from the hospital to the patient being remarkably close and being able to operate faster than a van. Therefore, with patients triaged at any level, emergency motorcycles have a faster reach, and thus are a better choice in time-sensitive scenarios.^30,31^

During the outbreak of the coronavirus disease 2019 (COVID-19), the EMS situation was varying from any countries. The factors included the number of infected, social distancing measures, work from home policy, and the weather condition resulting in effects on the length of access to the patient.^32^ Present to future, the innovation applied used in EMS may aid EMS personnel in the prehospital situation.^33^

All motorcycle ambulance operations were not duty to delivery of patients due to the design of vehicles, including Thailand’s laws. However, the best model of motorcycle use in EMS is simply to bring medical equipment and personnel in to assess and treat emergency patients. After that, a traditional ambulance was used to deliver patient from the scene to the hospital. In out-of-hospital cardiac arrest patients, the use of a motorcycle ambulance with an AED will increase the chance of patient survival.^7^

## Limitations

This study had several limitations. First, the data came from only one source, a single EMS at Srinagarind Hospital. This means the characteristics of the population studied could be different from other units. Second, there are several factors affecting the response time, such as distance to reach the patient, preparedness of EMS personnel, dispatch time, and climate conditions. In this study, only some factors were studied, and in different units, various other contributing factors may result in different response times. Third, this study demonstrated the statistical significance between red categorized patients and operation time from 6:00am-9:00am and 3:00pm-6:00pm; however, that did not demonstrate the clinical significance because this study did not demonstrate the outcome of patients accessed by motorcycle compared with van ambulance in the red category, especially with suspected cardiac arrest. Fourth, this study did not demonstrate the type of patients or the number of patients on scene, which may affect the use of motorcycles in EMS operation. The problems which the authors addressed were the major trauma patients or multiple patients at the scene, and the motorcycle ambulance cannot operate in that situation. Finally, this study was carried out during the COVID-19 pandemic, which may have caused traffic conditions to differ from normal times, which also can affect the response time.

## Conclusions

The motorcycle ambulance was a new style of EMS vehicle designed to shorten response time, particularly in cities with rush hour traffic or narrow streets. This study demonstrated the benefit of a motorcycle ambulance that is faster and more efficient in reaching emergency patients than van ambulances, especially at the operation times between 6:00am-9:00am and 3:00pm-6:00pm if the distance from the hospital to the scene is less than 10 kilometers.
